# Plasma lipidomic profiling in murine mutants of Hermansky–Pudlak syndrome reveals differential changes in pro- and anti-atherosclerotic lipids

**DOI:** 10.1042/BSR20182339

**Published:** 2019-02-19

**Authors:** Jing Ma, Raoxu Wang, Sin Man Lam, Chang Zhang, Guanghou Shui, Wei Li

**Affiliations:** 1 Beijing Key Laboratory for Genetics of Birth Defects, Beijing Pediatric Research Institute; Genetics and Birth Defects Control Center, National Center for Children’s Health; MOE Key Laboratory of Major Diseases in Children; Beijing Children’s Hospital, Capital Medical University, Beijing 100045, China; 2 Institute of Genetics and Developmental Biology, Chinese Academy of Sciences, Beijing 100101, China

**Keywords:** atherosclerosis, Hermansky-Pudlak syndrome, lipidomics, lipid metabolism, vesicle trafficking

## Abstract

Atherosclerosis is characterized by the accumulation of lipid-rich plaques in the arterial wall. Its pathogenesis is very complicated and has not yet been fully elucidated. It is known that dyslipidemia is a major factor in atherosclerosis. Several different Hermansky–Pudlak syndrome (HPS) mutant mice have been shown either anti-atherosclerotic or atherogenic phenotypes, which may be mainly attributed to corresponding lipid perturbation. To explore the effects of different HPS proteins on lipid metabolism and plasma lipid composition, we analyzed the plasma lipid profiles of three HPS mutant mice, *pa* (*Hps9*^−/−^), *ru* (*Hps6*^−/−^), *ep* (*Hps1*^−/−^), and wild-type (WT) mice. In *pa* and *ru* mice, some pro-atherosclerotic lipids, e.g. ceramide (Cer) and diacylglycerol (DAG), were down-regulated whereas triacylglycerol (TAG) containing docosahexaenoic acid (DHA) (22:6) fatty acyl was up-regulated when compared with WT mice. Several pro-atherosclerotic lipids including phosphatidic acid (PA), lysophosphatidylserine (LPS), sphingomyelin (SM), and cholesterol (Cho) were up-regulated in *ep* mice compared with WT mice. The lipid droplets in hepatocytes showed corresponding changes in these mutants. Our data suggest that the *pa* mutant resembles the *ru* mutant in its anti-atherosclerotic effects, but the *ep* mutant has an atherogenic effect. Our findings may provide clues to explain why different HPS mutant mice exhibit distinct anti-atherosclerotic or atherogenic effects after being exposed to high-cholesterol diets.

## Introduction

Atherosclerosis is a common and important pathological factor of cardiovascular disease. It is characterized by the accumulation of lipid-rich plaques on the arterial wall followed by intraplaque hemorrhage, plaque rupture, thrombosis, calcification, the formation of neoplasia, and stenosis of the vessel lumen causing severe ischemia and necrosis of tissues and organs, and even death [1,2]. The pathogenesis of atherosclerosis is very complex and has not yet been fully elucidated. Dyslipidemia, obesity, hypertension, diabetes, and other factors are known to be associated with the development of atherosclerosis [2–5].

The human body maintains the metabolic homeostasis of lipids through precise regulatory mechanisms. Once the lipid homeostasis is disturbed, arterial lesions may occur. Therefore, an in-depth study of the metabolic processes of lipids will contribute to a better understanding of the pathogenesis of atherosclerosis. Hepatocytes are the main sites of lipid metabolism, and play an important role in lipid metabolism. The lipid metabolism process in hepatocytes directly affects the content of various lipids in plasma [6,7]. The formation and fusion of lipid droplets and the transport, secretion, and degradation of lipids in hepatocytes are dependent on intracellular vesicle transport [8,9].

Many studies in recent years have shown that several Hermansky–Pudlak syndrome (HPS) [10] proteins are involved in the vesicular transport of intracellular cargoes to lysosomes and lysosome-related organelles (LROs) [11,12]. HPS is characterized by oculocutaneous albinism, bleeding tendency, and ceroid deposition in several tissues. HPS may cause lung fibrosis, colitis, and cardiomyopathy in some cases [13,14]. The main pathogenesis of HPS is the disrupted biogenesis and/or function of LROs, including melanosomes, Weibel–Palade bodies (WPB), and platelet-dense granules (DG) as well as secretory lysosomes [12,15–19]. Based on the important role of these HPS proteins in intracellular vesicle trafficking, we hypothesize that HPS proteins are likely to be involved in lipid metabolism in hepatocytes, thereby affecting lipid content in plasma. In one pilot study, five genetically distinct HPS mutants were studied for this purpose. After consuming an atherogenic diet for 14 weeks, *Hps6* mutant mice (ruby-eye, *ru*) had significantly less atherosclerotic lesions than the wild-type (WT) control. In contrast, *Hps1* mutant mice (pale ear, *ep*) had lesions similar to WT animals. After 48 weeks, *ru* mice showed greater than 50% survival. In contrast, no animals from the *ep* mice or the WT C57BL/6 strains survived. Even before 40 weeks, the survival rate of *ep* mice was lower than that of the WT control group [20]. These data implied that different HPS proteins play different roles in lipid metabolism.

In the present study, the plasma lipid profiles of HPS mutant mice were characterized by plasma lipidomics and their accumulation of lipid droplets in hepatocytes was observed after overnight starvation. Our results suggested that different HPS proteins may play different roles in lipid metabolism, conferring protective or accelerating effects on atherosclerosis.

## Materials and methods

### Mice and plasma sample collection

The *pa, ru*, and *ep* mutant mice (*pa*, HPS9 deficient [21], *ru*, HPS6 deficient [22], *ep*, HPS1 deficient [23]) and the WT C57BL/6J mice were originally obtained from The Jackson Laboratory (Maine, U.S.A.) and were maintained in Dr Richard T. Swank’s laboratory. All these mutants arose from spontaneous mutations in the C57BL/6J background. These mice were bred at the animal facility of the Institute of Genetics and Developmental Biology (IGDB), Chinese Academy of Sciences. All animal protocols were approved by the Institutional Animal Care and Use Committee of IGDB. The mutant mice were maintained as homozygotes through intercrosses of heterozygotes. The genotypes of these mutants were confirmed by PCR genotyping methods based on the nature of the mutations [24].

One year old male mice with chow diet were used. A 30-μl aliquot of tail blood of each mouse was collected with 1/10 volume of sodium citrate as anticoagulant and was subsequently centrifuged (4000×***g*** for 10 min) to collect the plasma. The plasma samples were then transferred into sterile tubes and immediately stored at −80°C until further analysis.

### Lipidomics profiling of plasma

Lipids were extracted from plasma (20 µl) using a modified Bligh and Dyer’s extraction procedure (double rounds of extraction) and dried in the SpeedVac [25]. Lipidomic analyses were carried out on an Exion UPLC system coupled with a QTRAP 6500 PLUS system (Sciex) as described previously [26,27]. In brief, polar lipids were separated on a Phenomenex Luna Silica 3 µm column (i.d. 150 × 2.0 mm) under the following chromatographic conditions: mobile phase A (chloroform:methanol:ammonium hydroxide, 89.5:10:0.5) and mobile phase B (chloroform:methanol:ammonium hydroxide:water, 55:39:0.5:5.5) at a flow rate of 270 µl/min and column oven temperature at 25°C. Individual polar lipid species were quantitated by referencing to spiked internal standards including phosphatidyl choline (PC)-14:0/14:0, d31-PC16:0/18:1, phosphatidyl ethanolamine (PE)-14:0/14:0, d31-PE-16:0/18:1, phosphatidyl serine (PS)-17:0/20:4, d31-PS-16:0/18:1, phosphatidic acid (PA)-17:0/17:0, PA-17:0/20:4, phosphatidyl glycerol (PG)-14:0/14:0, d31-PG-16:0/18:1 glucosylceramide (GluCer)-d18:1/8:0, ceramide (Cer)-d18:1/17:0, C14:0-lysobisphosphatic acid (LBPA), d31-phosphatidyl inositol (PI)-16:0/18:1, sphingosine-1-phosphate (S1P)-d17:1, sphingolipids (Sph)-d17:1, sphingomyelin (SM)-d18:1/12:0, lyso-phosphatidylserine (LPS)-17:0, LPC-17:0, LPE-17:0, LPI-17:0, LPA-17:0 obtained from Avanti Polar Lipids (Alabaster, AL, U.S.A.) and PI-8:0/8:0 from Echelon Biosciences, Inc. (Salt Lake City, UT, U.S.A.). GM3 species were quantitated using GM3 d18:1/17:0 synthesized in-house as an internal standard. All polar lipids were analyzed in the ESI mode. PC, Cer, GluCer, SM, S1P, and Sph were detected in the positive ion mode, while remaining polar lipids were detected in the negative ion mode.

Glycerol lipids including diacylglycerols (DAG) and triacylglycerols (TAG) were quantitated using a modified version of reverse-phase HPLC/MRM in the ESI-positive ion mode [28]. Separation of neutral lipids were achieved on a Phenomenex Kinetex-C18 2.6 µm column (i.d. 4.6 × 100 mm) using an isocratic mobile phase containing chloroform:methanol: 0.1 M ammonium acetate, 100:100:4 (v/v/v) at a flow rate of 170 µl for 17 min. Levels of short-, medium-, and long-chain TAGs were calculated by referencing with spiked internal standards of TAG(14:0)3-d5, TAG(16:0)3-d5, and TAG(18:0)3-d5 obtained from CDN isotopes, respectively. DAGs were quantitated using d5-DAG16:0/16:0 and d5-DAG18:1/18:1 as internal standards (Avanti Polar Lipids).

Free cholesterols (Cho) and cholesteryl esters (CEs) were analyzed in the atmospheric pressure chemical ionization (APCI) positive ion mode as described previously with d6-Cho and d6-C18:0 CE (CDN isotopes) as internal standards [29].

### Oil Red O staining

The WT, *pa, ru*, and *ep* mutant mice were starved for 12 h and their livers were removed and frozen in liquid nitrogen. The lipid droplets of livers were stained with Oil Red O. Briefly, 5–10 μm cryosectioned livers were fixed in ice-cold 10% formalin for 5–10 min, rinsed with distilled water for three-times, and stained with Oil Red O (Sigma, Cat# O0625-25G) solution (0.5% Oil Red O in propylene glycol) for 8–10 min in room temperature for 15 min. Then the slides were differentiated in 85% propylene glycol solution for 2–5 min and rinsed with distilled water for two-times. Nuclei were stained with Mayer’s Hematoxylin for 30 s, washed in running tap water, and mounted in aqueous mounting medium. The images were obtained by Nikon microscope (model eclipse Ci-L) and Nikon microscope camera (model DS-Ri2).

### Statistical analysis

Results were expressed as the means ± S.E.M. The significance level of lipid classes was set at *P*<0.05 using Kruskal–Wallis test. Student’s *t* tests were used for the analysis of the lipid droplets after Oil Red O staining.

## Results

### Overall changes in plasma lipid compositions in HPS mutant mice

Three 12-month-old WT (C57BL/6J) mice or mutant mice with chow diet were selected for the present study. The tail blood of each mouse was taken and 1/10 volume of sodium citrate anticoagulant was added. After centrifuging for 10 min, the upper plasma was used to perform the lipidomic profiling. Overall, more than 20 classes of lipids including CEs, GluCer, LPE, Cho, PE, TAGs, DAGs, Cer, LPS, PA, and PI were detected (Table 1).

**Table 1:** An overview of the changes in various lipid compositions in different mutants compared with WT mice

Lipid class	WT	*pa*	*ru*	*ep*
**CE**	0.00047	0.000542*	0.00054*	0.000532*
**Cho**	0.000176	0.000193	0.000211	0.000216*
**TAG**	0.000144	0.000127	0.000168	0.000209*
**DAG**	4.27E-05	3.68E-05*	3.84E-05*	4.25E-05
**Acylcarnitine**	4.54E-07	5.79E-07	6E-07	5.28E-07
**PC**	0.000473	0.000472	0.000543	0.00054
**PE**	1.37E-05	1.72E-05	1.88E-05*	2.12E-05
**PA**	2.75E-07	2.81E-07	2.95E-07	3.63E-07*
**PI**	2.64E-05	2.52E-05	2.34E-05*	2.57E-05
**PS**	2.39E-06	2.33E-06	2.3E-06	2.33E-06
**PG**	2.28E-07	2.49E-07	2.29E-07	2.56E-07
**LBPA**	1.72E-06	2E-06	1.92E-06	1.55E-06
**LPC**	0.000134	0.000143	0.000158	0.00014
**LPE**	9.82E-07	1.37E-06	1.95E-06*	1.48E-06*
**LPI**	2.19E-07	2.42E-07	2.81E-07	2.29E-07
**LPS**	1.5E-07	1.38E-07	1.48E-07	1.72E-07
**SM**	2.21E-05	2.01E-05	2.35E-05	2.51E-05
**Cer**	1.51E-06	1.03E-06	1.23E-06	1.42E-06
**GluCer**	4.94E-07	6.86E-07	7.43E-07	7.3E-07*
**LacCer**	9.7E-09	1.25E-08	1.41E-08	1.33E-08
**S1P**	2.38E-08	2.37E-08	2.59E-08	2.69E-08

*P<0.05.

**Figure 1:**
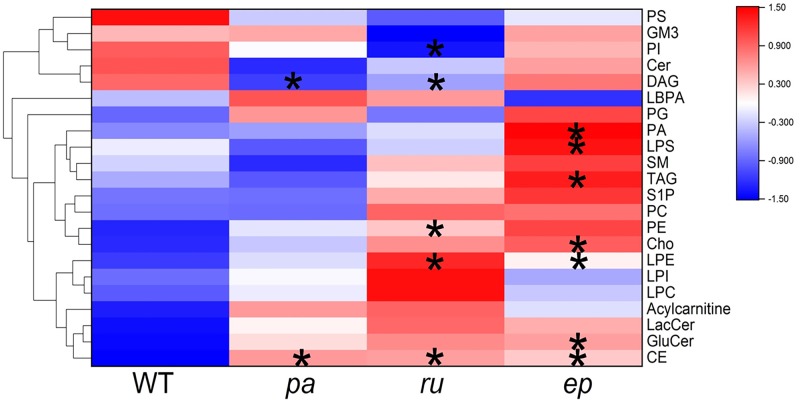
The heatmap of the changes in the various lipid classes in different mutants compared with WT mice *P<0.05.

The changes in the plasma lipid profiles for various mutants together with WT mice were analyzed and the overall changes are shown in Figure 1. In particular, we focussed on the lipid classes with statistically significant changes in the mutants compared with the WT mice. Of 22 classes of lipids measured, DAG was reduced and CE was increased in *pa* mice. PI and DAG were reduced, while PE, LPE, and CE were increased in *ru* mice. PA, LPS, TAG, Cho, LPE, GluCer, and CE were increased in *ep* mice (Figure 1).

**Figure 2:**
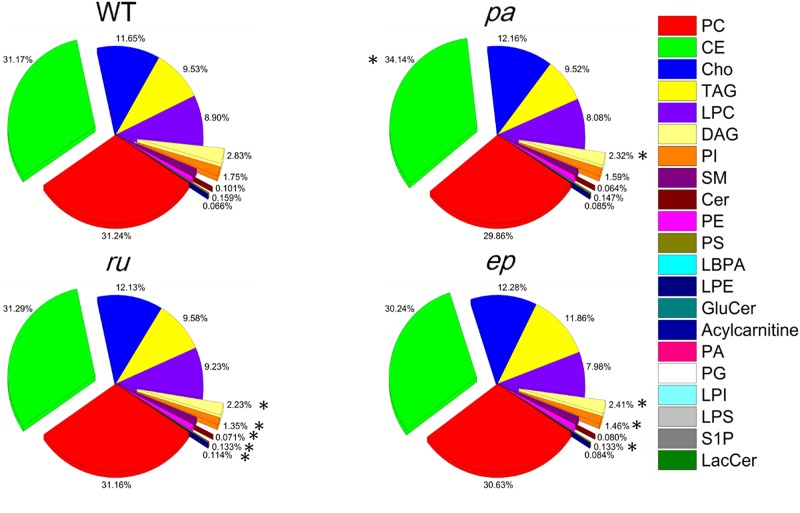
Pie chart showing the proportions of different plasma lipids for WT and mutant mice *P<0.05.

The data in Figure 1 provide an overview of the changes in various lipid compositions in the plasma of different HPS mutants. The proportion of every single lipid class detected in terms of total lipids was further calculated for the mutant and WT mice as shown in Figure 2. The most abundant lipids in plasma were CE and PC followed by Cho, TAG, and LPC. In three mutant mice, the proportion of the above mentioned lipids did not change significantly compared with those in the WT mice. Only the proportion of CE in *pa* mice significantly increased. For those lipids found in small proportions, the DAG ratio was reduced in all three mutants. In *ru* mice, the proportion of PI, Cer, and PS was significantly decreased, while the proportion of LPE was significantly increased. Moreover, the PI and PS ratios were significantly decreased in *ep* mice. The reduced proportion of Cer in *ru* mice may be responsible for the distinct phenotypes observed between the *ru* and *ep* mice.

### Changes in Cer

**Figure 3:**
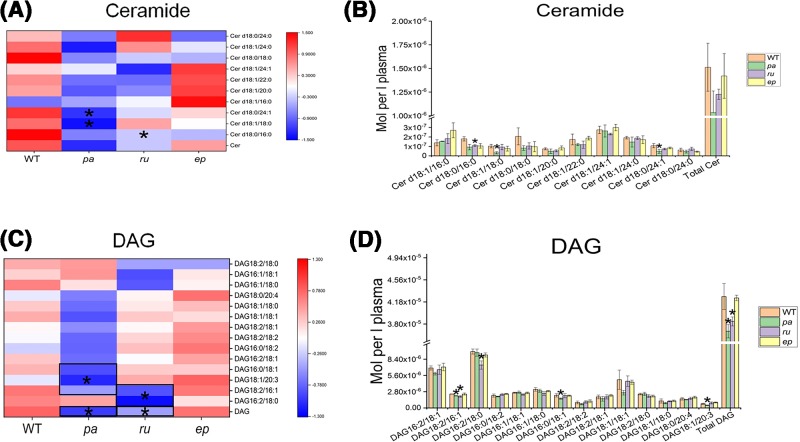
Cer and DAG profiling in HPS mutant mice The changes in Cer (**A**,**B**) and DAG (**C**,**D**) in *pa, ru*, and *ep* mutants compared with WT mice. *P<0.05.

In our study, there was no significant change in total plasma Cer amongst the three mutant mice compared with the WT mice, but some special species of Cer, Cer d18:0/24:1, Cer d18:1/18:0, were significantly reduced in the *pa* group, and Cer18:0/16:0 was significantly reduced in the *ru* group (Figure 3A,B). Ceramides have been shown to be associated with apoptosis [30,31] and promote apoptosis of a variety of cells, including endothelial cells. Endothelial cell apoptosis is recognized as an early event in the development of atherosclerosis. Several studies have recently found that Cer is associated with atherosclerosis. ApoE-deficient mice, a well-known atherosclerotic model, have significantly increased plasma lipids after exposure to cigarettes, including Cho, ceramides, and cerebrosides [32]. Inhibition of the enzyme involved in sphingolipid biosynthesis in ApoE-deficient mice reduced plasma levels of SM, Cer, and S1P, resulting in a significant reduction in atherosclerotic lesions [33]. The decrease in Cer18:0/16:0 in *ru* mice compared with WT may be related to the atherosclerosis-resistant property in the mutant mice. This result was in agreement with the decrease in the proportion of Cer in *ru* mice, further supporting the potential atherosclerosis-protective effect in this mutant.

### Changes in DAG

In *pa* and *ru* mice, the total DAG was significantly decreased. In particular, DAG18:2/16:1 and DAG16:2/18:0 were significantly deceased in the *ru* group, and DAG18:2/16:1, DAG16:0/18:1, and DAG18:1/20:3 were significantly deceased in the *pa* group (Figure 3C,D).

DAG is a well-known allosteric activator of protein kinase C (PKC). DAG has been shown to activate specific PKC isoforms whose activation were linked with insulin resistance (IR) [34–36]. Additionally, DAG is also associated with hypertension. DAG containing palmitic acid (16:0) was demonstrated to be genetically correlated in a statistically significant manner with hypertension [37]. Hypertension, abnormal blood sugar, and obesity are all closely related to the pathogenesis of atherosclerosis. Therefore, the decrease in DAG18:2/16:1 and DAG16:2/18:0 in *ru* mice may be involved in its anti-atherosclerosis effect.

### Changes in LPS, PA, and Cho

**Figure 4:**
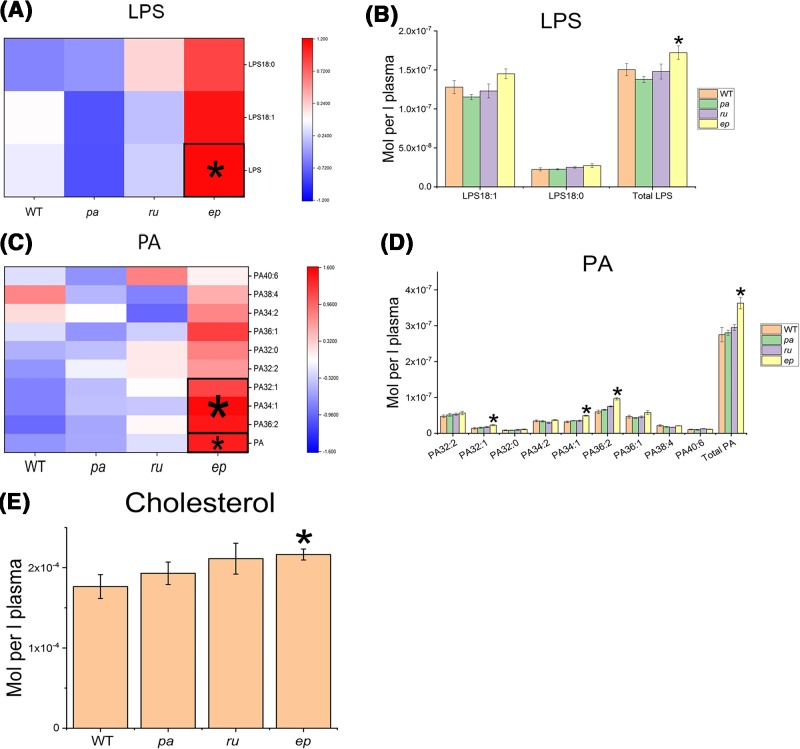
LPS, PA and Cho profiling in HPS mutant mice The changes in LPS (**A**,**B**), PA (**C**,**D**), and Cho (**E**) in *pa, ru*, and *ep* mutants compared with WT mice. *P<0.05.

The total LPS level was specifically increased in *ep* mice compared with WT mice (Figure 4A,B). LPS promotes platelet activation and the formation of foam cells. Both platelet activation and the formation of foam cells contribute to the progression of atherosclerosis [38]. LPS also plays an important role in the inflammatory response. LPS treatment can promote chemotactic migration of various cells such as mouse fibroblasts, human glioma cells, and some human leukemia THP-1 cells, thereby promoting atherosclerosis [39].

The total PA level was specifically increased in *ep* mice, especially for PA32:1, 34:1, and 36:2 (Figure 4C,D). Macrophages and dendritic cells are specialized phagocytic and antigen-presenting cells that provide immune surveillance and bridge the innate and adaptive immune system. Particular antigens are engulfed by phagocytosis, while soluble antigens are internalized by macropinocytosis. On the plasma membrane of macrophages and immature dendritic cells, the content of PA increased. PA has been shown to promote actin polymerization by increasing the concentration of monomeric actin and controlling the cleavage of filaments. Actin polymerization has a driving effect on the phagocytosis and macropinocytosis of the above antigens [40]. There was evidence that PA increases the uptake of low-density lipoprotein (LDL) by cells [41]. Furthermore, PA can also stimulate NADPH oxidase and partially increases reactive oxygen species induced by high glucose levels [42].

The Cho level was increased in *ep* mice compared with WT mice (Figure 4E). Hypercholesterolemia is a major risk factor for atherosclerosis, and drug treatment that lowers plasma cholesterol levels can reduce cardiovascular morbidity. Aggregated LDL is absorbed by macrophages, resulting in cellular cholesterol accumulation and foam cell formation [43]. A sustained increase in plasma cholesterol levels is associated with increased cholesterol deposition in the intima, which initiates and promotes the progression of atherosclerosis [44]. Therefore, the elevation of LPS, PA, and Cho in the plasma may put *ep* mice at high risk of developing atherosclerosis.

### Changes in TAG

**Figure 5:**
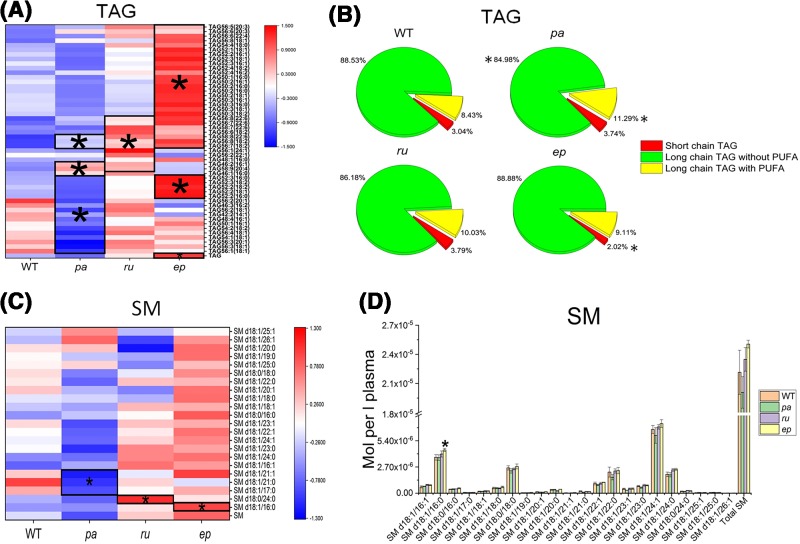
TAG and SM profiling in HPS mutant mice The changes in TAG (**A**,**B**) and SM (**C**,**D**) in *pa, ru*, and *ep* mutants compared with WT mice. *P<0.05.

Our results showed that the total TAG level was significantly increased in the *ep* group. In *ru* mice, although no significant change in total TAG was observed, TAG species with a fatty acid chain containing docosahexaenoic acid (DHA) (22:6) were increased (Figure 5A,B). It was reported that fats containing saturated fatty acids of chain length 12:0–16:0 increased the serum total cholesterol and LDL-cholesterol (LDL-C), while the monounsaturated fatty acids (MUFA) and polyunsaturated fatty acids (PUFA) reduce the LDL-C level [45]. The increase in plasma total cholesterol and LDL-C is one of the important pathogenic factors of atherosclerosis [45].

Similarly, IR and inflammation are also potential pathogenic factors of atherosclerosis. TAG species containing saturated or monounsaturated fatty acids correlate positively with IR whereas TAGs containing essential fatty acids correlate negatively with IR [46]. Polyunsaturated omega-3 fatty acids (n-3, PUFA), eicosapentaenoic acid (EPA, C20:5 n-3), and DHA (C22:6 n-3) have been proven to exhibit a protective effect on heart disease and can reduce inflammation. The inflammatory response of endothelial cells plays an important role in the initiation and development of atherosclerosis [47,48]. Studies by Toborek et al. [49] demonstrated that linolenic acid stimulated the transcriptional activity of NF-κB and activator protein 1, and significantly enhanced the mRNA level of the inflammatory mediators, such as tumor necrosis factor, monocyte chemoattractant protein 1, vascular cell adhesion molecule 1, and intercellular adhesion molecule 1. By comparison, oleic acid and the n-3 fatty acid DHA were demonstrated to inhibit cytokine-induced expression of VCAM-1 and other indices of endothelial activation in cultured cells [50].

The increase in TAG containing a DHA (22:6) side chain in *ru* mice may contribute to the atherosclerosis resistance effect observed in these mice. The increase in TAG in *ep* mice, especially TAG containing saturated or monounsaturated fatty acids, may relate to an atherogenic phenotype. Several lipids containing a MUFA or a saturated fatty acid are decreased in the *pa* group, although there is no significant change compared with the WT group in terms of the total TAG. These observations suggest that the distinct effects of various HPS mutants are not only related to the total TAG levels but also to the fatty acid chain length and the degree of unsaturation.

### Changes in SM

Our study uncovered significant elevation of SM d18:1/16:0, a major species of SM, in *ep* mice compared with WT mice. While SM d18:1/17:0, d18:1/21:1, and d18:1/21:0 were decreased in the *pa* group (Figure 5C,D). Studies have shown that short- and medium-chain fatty acid SMs are positively correlated with intra-abdominal fat and insulin [51]. An increase in sphingolipids can reduce the reverse cholesterol transport, thereby increasing the risk of hyperlipidemia-related diseases [52]. Some specific lipid substances such as SM, Cer, and glycosphingolipid may have an atherogenic effect [32]. An increase in SM d18:1/16:0 in *ep* mice may be associated with the pro-atherosclerosis effect observed in these mice.

In summary, the lipidomic profiles of three HPS mutant mice (*pa* (*HPS9*^−/−^), *ru* (*HPS6*^−/−^), and *ep* (*HPS1*^−/−^)) showed that Cer and DAG were reduced in *pa* and *ru* mice, compared with WT mice, while TAG (containing DHA) was increased. In contrast, PA, LPS, TAG, SM, and Cho were elevated in *ep* mice compared with WT mice (Table 2).

**Table 2:** The different changes in plasma lipids observed in *pa, ru*, and *ep* mice compared with WT mice

*pa*	*ru*	*ep*
Cer ↓	Cer ↓	PA ↑
DAG ↓	DAG ↓	LPS ↑
TAG (DHA) ↑	TAG (DHA) ↑	TAG ↑
SM ↓		SM ↑
		Cho ↑

### The lipid droplets in liver

**Figure 6:**
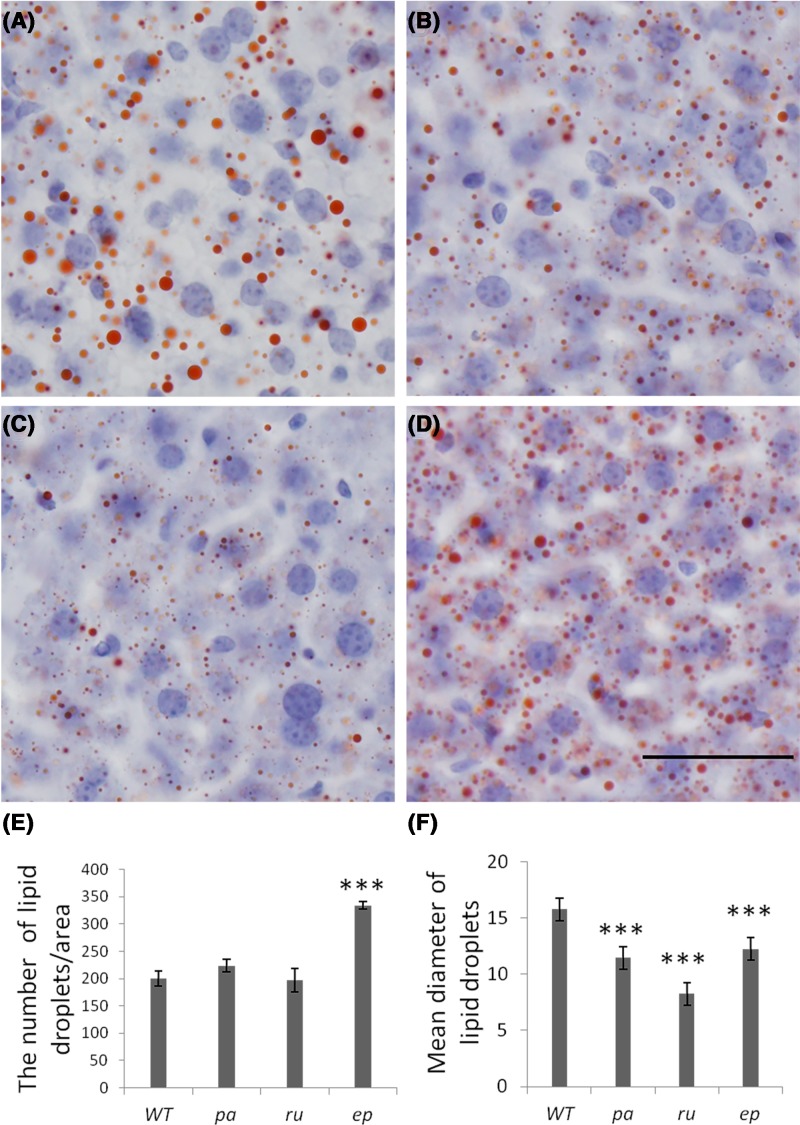
Morphological changes of lipid droplets in the liver of HPS mutant mice Oil Red O staining of lipid droplets in the liver cells of (**A**) WT, (**B**) *pa*, (**C**) *ru*, and (**D**) *ep* mutant mice. Scale bar: 25 μm. (**E**) Quantitation analysis of the numbers of lipid droplets per area. The images were analyzed with NIH ImageJ software; n=9, ***P<0.001. (F) Quantitation analysis of the mean diameters of lipid droplets. The total number of lipid droplets analyzed: 2388 lipid droplets from WT mice, 2744 lipid droplets from *pa* mice, 2079 lipid droplets from *ru* mice, 4102 lipid droplets from *ep* mice, ***P<0.001.

Hepatocytes play a central role in lipid metabolism and are the major sites for the production of plasma lipoproteins. We stained the frozen sections of liver tissues of WT and the three mutant mice by Oil Red O staining. Figure 6 shows a representative picture of the lipid droplets (red) in the liver cells of each mouse. The results showed that there was no significant change in the number of lipid droplets in the *pa* and *ru* mice, while the number of lipid droplets in the *ep* mice significantly increased compared with that of WT mice. The diameter of the lipid droplets was significantly reduced in all the three mutant mice. Especially, the mean diameter of lipid droplets is the smallest in *ru* mice. This indicated that in these three mutant mice, the lipid metabolism in the liver has different degrees of abnormality, suggesting that these three HPS proteins play certain functions during the formation or fusion of lipid droplets.

## Discussion

In recent years, more and more studies have shown that various lipids in plasma are directly related to the initiation and progression of atherosclerosis. Amongst them, multiple lipid classes have different atherosclerosis-associated effects, such as Cer, DAG, SM, PA, LPS, and Cho, and they promote the occurrence and development of atherosclerosis in various ways. Briefly, LPS and Cho promote foam cell formation and thrombosis [38,43]. PA promotes phagocytosis of macrophages and dendritic cells and increases the uptake of LDL [40,41]. Cer promotes endothelial cell apoptosis [30,31]. TAG, DAG, and SM all promote IR [35,36,46,51]. Notably, various TAG species have different effects on atherosclerosis. TAGs containing saturated and monounsaturated fatty acid side chains have an atherogenic effect, while TAGs containing PUFA side chains have an anti-atherosclerosis effect [45].

The underlying mechanism of different anti-atherosclerotic phenotypes of HPS mutants after stimulation with a high-cholesterol diet is unknown [20]. The different changes in plasma lipids observed in *ru* and *ep* mice are consistent with their phenotypes that promotes or resists atherosclerosis (Table 2). This provides a possible explanation for the differential anti-atherosclerotic effects exhibited by different HPS mice after stimulation with a high-cholesterol diet.

There is no direct evidence for the correlation of *pa* mutant with atherosclerosis. However, in a study of melanosomes, HPS9 and HPS6 were shown to act in the same pathway during melanogenesis [53]. It can be seen from our lipidomic results that Cer, DAG, TAG, and SM were reduced in *pa* mice compared with WT mice whereas TAG (containing DHA) was increased (Table 2). These plasma lipid changes in *pa* mice are similar to those in *ru* mice. The similar results from the Oil Red O staining of liver from *pa* and *ru* mice are additional support to their similar effects on lipid metabolism. Therefore, we speculate that *pa* mice may have an anti-atherosclerotic phenotype upon an atherogenic diet. However, this speculation requires future investigation.

Additionally, atherosclerosis is considered to be associated with NAFLD and IR. The increase in PA, LPS, TAG, SM, and Cho in the plasma of *ep* mice and the phenotype of hepatic lipid droplets accumulation may also indicate its role in promoting atherosclerosis. Therefore, our results provide an early warning to HPS patients. For example, HPS1 patients should pay more attention to diet in an effort to avoid the risk of atherosclerosis caused by a high-fat diet. It also suggests that the early monitoring of plasma lipid levels is very important to prevent cardiovascular disease.

Our results also indicate that different HPS proteins perform different functions in the process of lipid metabolism. The main site of lipid metabolism is in hepatocytes, where lipids are mainly stored in lipid droplets, which are independent organelles that include a lipid-rich core and surrounding phospholipid monolayers. The lipid droplets are generated from the phospholipid bilayer of the endoplasmic reticulum and are formed by budding. Their synthesis and transport are complex processes that involve many proteins. Carboxylesterase 3 and CIDEB (cell death-inducing DNA fragmentation factor-like effector B) are known to be involved in the transport of lipids from lipid droplets to VLDL [7,8,54]. Lipids in lipid droplets can also be degraded by lysosomal lipases through autophagy [9]. However, little is known about the molecular mechanism involved in these processes.

Experimental results of Oil Red O staining in the three mutant mice showed that the mean diameter of lipid droplets decreased in the hepatocytes of *pa* and *ru* mice. While the number of lipid droplets significantly increased and the mean diameter of lipid droplets significantly decreased in the hepatocytes of *ep* mice. Since these HPS proteins are involved in the vesicle trafficking process from endosomes to lysosomes and lysosomal-associated organelles, these three HPS proteins may be involved in the formation or fusion of the lipid droplets. According to the lipid droplet phenotypes, we speculated that HPS9 and HPS6 may play some role in the formation of lipid droplets or lipids transportation toward lipid droplets. Loss of HPS9 or HPS6 may lead to the formation of smaller lipid droplets. HPS1 may play a role in the fusion of lipid droplets, and may also be involved in lipids sorting out from lipid droplets or lipids degradation through autophagy. The underlying mechanism requires further study. Moreover, these results also imply that other HPS proteins may also perform certain functions in lipid metabolism.

## Abbreviations

CE: cholesteryl ester
Cer: ceramide
Cho: cholesterol
DAG: diacylglycerol
DHA: docosahexaenoic acid
GluCer: glucosylceramide
HPS: Hermansky–Pudlak syndrome
IR: insulin resistance
LacCer: Lactosylceramide
LDL: low density lipoprotein
LDL-C: LDL-cholesterol
LPS: lysophosphatidyl serine
LRO: lysosome-related organelle
MUFA: monounsaturated fatty acid
PA: phosphatidic acid
PC: phosphatidyl choline
PE: phosphatidyl ethanolamine
PG: phosphatidyl glycerol
PI: phosphatidyl inositol
PS: phosphatidyl serine
PUFA: polyunsaturated fatty acid
SM: sphingomyelin
S1P: sphingosine-1-phosphate
TAG: triacylglycerol
WT: wild-type
